# Rosiglitazone use and associated adverse event rates in Canada: an updated analysis

**DOI:** 10.1186/s13104-015-1448-6

**Published:** 2015-09-29

**Authors:** Sandra Iczkovitz, Daniella Dhalla, Jorge A. Ross Terres

**Affiliations:** Medical Affairs, GlaxoSmithKline Inc., 7333 Mississauga Road, Mississauga, ON L5N 6L4 Canada; GlaxoSmithKline Inc., 2301 Renaissance Boulevard, King of Prussia, PA 19406 USA

**Keywords:** Rosiglitazone, Utilization, Adverse events, Risk communication

## Abstract

**Background:**

We previously reported on the change in the use of rosiglitazone-containing products (RCP) and adverse event reporting rates in Canadian patients between 2004 and 2010. The present study extends this analysis to include the January 2011 to December 2012 time period.

**Methods:**

RCP utilization rates were obtained from IMS Health Brogan’s longitudinal de-identified patient database, LRx. GlaxoSmithKline’s global adverse events database was used to extract adverse events (AE), serious adverse events (SAE), and cardiac adverse events (CAE) reported in Canadian patients receiving RCP from April 2004 to December 2012. The patient utilization information from the LRx database was used to estimate rates per 100,000 patients.

**Results:**

An estimated 182,841 patients were dispensed RCP prescriptions between April 2004 and December 2012. The total number of patients using RCP decreased by 85 % from 2011 to 2012. From its peak use in 2007, the number of patients filling a prescription decreased 97 %. A total of 1069 AEs were reported during the study period, of which 32 AE’s were reported from Jan 2011 to Dec 2012. The average monthly reporting rates of AE’s, SAE’s and CAE’s over 2011–2012 were 10.8/100,000 patients, 9.1/100,000 patients and 5.0/100,000 patients, respectively.

**Conclusions:**

The utilization of RCP in Canada has significantly declined. The significance of the adverse event rate information presented is uncertain and must be evaluated within the context of the well known factors that can influence AE reporting rates, as well as limitations to the methods used to estimate these reporting rates.

## Background

Rosiglitazone, a member of the thiazolidinedione class of oral anti-diabetic agents, improves glycemic control in patients with type 2 diabetes mellitus by improving insulin sensitivity, decreasing insulin resistance and lowering blood glucose levels. In May 2007, the publication of a meta-analysis evaluating the effect of rosiglitazone on cardiovascular morbidity and mortality generated significant public attention. The analysis found rosiglitazone treatment was associated with a statistically significant increased risk of myocardial infarction (OR 1.43, CI 1.03–1.98) in comparison with the use of placebo or other anti-diabetic agents [1].

Results from Rosiglitazone Evaluated for Cardiovascular Outcomes and Regulation of Glycemia in Diabetes (RECORD), a long term cardiovascular outcomes trial, became available in 2009 [2]. In contrast with the findings of the 2007 meta-analysis, this trial’s results found no increased risk of cardiovascular death, myocardial infarction or stroke associated with RCP use compared with standard glucose-lowering drugs in people with type 2 diabetes mellitus [2].

At the request of the United States (US) Food and Drug Administration (FDA), an independent comprehensive, expert re-evaluation of the RECORD data was conducted by the Duke Clinical Research Institute (DCRI). The readjudicated results assessed rosiglitazone versus the standard-of-care diabetes drugs metformin and sulfonylurea and confirmed the original RECORD findings [3]. In November 2013, after an extensive review of the RECORD trial and other data, the FDA concluded that there is no statistically significant difference between rosiglitazone and older diabetes drugs with respect to the risk of death or major adverse cardiovascular outcomes, other than the known class effect of heart failure [4]. This conclusion led the FDA to require GlaxoSmithKline (GSK) to remove the boxed warning on myocardial infarction in the US rosiglitazone label information, to substantially lift the restrictions on distribution of rosiglitazone-containing products, and to modify the US risk evaluation and mitigation strategy (REMS) program and Medication Guide.

In Canada, following publication of the 2007 meta-analysis, and before the results of RECORD were available and readjudicated, two Health Canada-endorsed risk communications were released [5, 6]. The first risk communication, in June 2007, provided information about the meta-analysis and discussed rosiglitazone benefit-risk treatment considerations [5]. The second risk communication, in November 2007, provided additional information and introduced restrictions on the use of AVANDIA^®^ (rosiglitazone), AVANDAMET^®^ (rosiglitazone and metformin), and AVANDARYL^®^ (rosiglitazone and glimepiride) [6].

Subsequent to the meta-analysis’ publication and these first two Health Canada-endorsed risk communications, several authors reported a marked decline in the utilization of RCP in Canada [7–10] These reports included an analysis by Rawson and Terres, which evaluated the impact of these risk communications on the change in use of RCP as monotherapy or as part of triple or triple-plus combination therapy between April 2004 and December 2010, as well as an analysis of the rates of overall adverse events (AEs), serious adverse events (SAEs) and cardiac AEs before and after the introduction of the November 2007 prescribing changes [9]. That publication noted that it was not possible to determine whether the decline in prescriptions was attributable to the publication of the meta-analysis, the changes in prescribing guidelines, media attention or a combination of these factors.

In November 2010, a third Health Canada-endorsed risk communication was released [11]. This communication introduced further restrictions on the use of RCP for the treatment of type 2 diabetes mellitus in Canada and required that physicians document the eligibility of patients as per rosiglitazone’s updated label, counsel patients on the benefits-risks of rosiglitazone treatment, including the cardiovascular risks, and obtain their written informed consent prior to starting or renewing a prescription for a RCP. Building upon the previous findings of Rawson and Terres, and as part of GSK’s ongoing regulatory commitment to Health Canada, the objective of the present study was to report on the change in RCP utilization patterns and reporting rates of AEs and cardiac AEs in Canada following this third risk communication.

## Methods

The methods for the present analysis are consistent with those described in a previous study on RCP utilization and adverse event rates in Canada [9]. Briefly, a cross-sectional ecological study in patients dispensed RCP in Canada was conducted. The study population was defined as patients dispensed RCP in Canada between April 1, 2004 and December 31, 2012, based on IMS Brogan Health’s longitudinal de-identified patient database, LRx. At the time of this updated analysis, the LRx database contained approximately 200 million prescriptions for over 20 million patients, representing a capture of 74 % of prescriptions nationally [12]. As per IMS Health Brogan’s practice when performing LRx drug utilization studies, patients with inconsistent gender or age labels, who had only one rosiglitazone prescription from a pharmacy, had unreasonably high numbers of prescriptions (i.e. the numbers of prescriptions dispensed were extremely high and inconsistent with typical treatment patterns), and patients who filled prescriptions at pharmacies that had not consistently reported data to IMS Health Brogan for the past 7 years were excluded. Proportional allocation methods established by IMS Brogan Health, which utilize an additional IMS Brogan Health data source, CompuScript, were employed to estimate RCP utilization in the overall Canadian population. The number of patients receiving any rosiglitazone product per calendar month was estimated. Overall RCP utilization per month, as well as its use in monotherapy, dual combination therapy, or triple-plus combination therapy was estimated.

Estimated rates of AEs, serious AEs (SAEs) and cardiac AEs per 100,000 Canadian patients for the analysis period of April 1, 2004 to December 31, 2012 were calculated using Canadian AEs reported to GSK’s global adverse event reporting database during this timeframe as the numerator and the updated IMS-derived number of rosiglitazone patients as the denominator. GSK’s global adverse event reporting database uses the Medical Dictionary for Regulatory Activities (MedDRA), which is a clinically validated international medical terminology dictionary used by regulatory authorities in the pharmaceutical industry, to classify adverse events [13, 14]. Adverse events reported to involve the cardiac system (e.g. myocardial infarction, cardiac failure, hypertension, pericardial effusion, cardiac operation) receive a MedDRA “cardiac disorder” system organ classification within GSK’s global adverse event database, and were reported as cardiac adverse events in the present study.

## Results

Of the 241,806 patients identified as having received at least one RCP prescription between April 1, 2004 and December 31, 2012, 58,965 met IMS Health Brogan’s exclusion criteria. Accordingly, an estimated 182,841 patients were dispensed RCP prescriptions over this time period. The extension of this analysis to December 2012 resulted in the inclusion of an additional 1905 patients from the 2004–2010 analysis, which reported on a total of 180,936 patients [9].

**Fig. 1:**
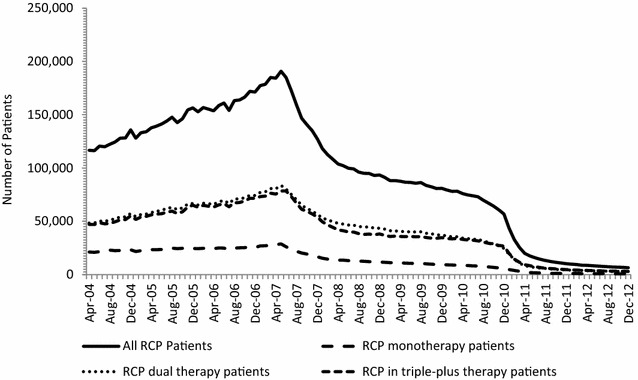
Number of patients receiving a rosiglitazone-containing product (RCP) by month, April 2004 to December 2012

The monthly utilization of RCP, overall and as monotherapy, dual combination therapy or triple-plus combination therapy, between April 2004 and December 2012 is presented in Fig. 1. The total number of patients dispensed a RCP prescription in Canada decreased by 85 %, from 43,774 patients in January 2011 to 6349 patients in December 2012. This represents a total decrease of 97 % from their peak utilization by 190,840 patients in May 2007. The decrease in RCP utilization was consistent across the treatment patterns studied, with rosiglitazone use as monotherapy, in dual therapy or triple-plus combination therapy falling by 84, 86 and 85 %, respectively, over the January 2011 to December 2012 time period, and by 97, 97 and 96 %, respectively, from their peak utilization in May 2007.

Between April 2004 and December 2012, a total of 1069 Canadian AE reports in RCP recipients were identified from GSK’s AE database, of which 32 AEs were reported in the January 2011 to December 2012 time period. The estimated monthly rates of AEs, SAEs and cardiac AEs, per 100,000 patients, for the April 2004 to October 2007, November 2007 to December 2010, and January 2011 to December 2012 time periods are provided in Table 1. Estimated monthly rates of AE’s declined from 13.1/100,000 patients in April 2004–October 2007 to 5.6/100,000 patients in November 2007–December 2010. The rate subsequently rose to 10.8/100,000 patients in January 2011–December 2012. A similar pattern of SAE’s and cardiac AE’s was observed throughout the three time periods.

**Table 1:** Estimated average monthly rates of adverse events per 100,000 patients in three time periods

	April 2004–October 2007			November 2007–December 2010			January 2011–December 2012		
	Average	Min/max	95 % CI	Average	Min/max	95 % CI	Average	Min/max	95 % CI
Adverse events	13.1	2.3/26.9	8.4–20.4	5.6	0.0/12.1	2.3–13.4	10.8	0.0/42.9	2.0–59.2
Serious adverse events^a^	5.9	0.6/21.1	3.1–11.3	3.3	0.0/8.6	1.1–10.4	9.1	0.0/42.9	1.3–62.4
Cardiac adverse events^b^	2.2	0.0/9.2	0.8–6.4	2.1	0.0/6.6	0.5–8.9	5.0	0.0/20.8	0.4–65

^a^Of the adverse events this row accounts for the serious adverse events

^b^Of the serious adverse events this row accounts for the cardiac adverse events

## Discussion

The results of this RCP utilization analysis show an 85 % decrease in RCP utilization between January 2011 and December 2012. This decline may be attributable to the November 2010 Health Canada-endorsed communication and the subsequent introduction of further restrictions on the use of RCP for the treatment of type 2 diabetes mellitus as well as the implementation of the requirement for patient informed consent. The overall Canadian decline in rosiglitazone utilization from May 2007 to December 2012 is similar to the findings of studies conducted in the United States, Europe and Australia [15–25].

The estimated adverse event reporting data must be considered in the context of several important limitations to the methodology employed to estimate these rates. In contrast to clinical trials where adverse events are proactively collected, passive AE reporting systems suffer from an indeterminate degree of reporting bias. Many factors can influence adverse event reporting rates, such as actual or anticipated litigation and publicity surrounding a type of event. These factors can cause under-reporting and/or over-reporting bias. Another limitation of passive AE reporting systems is the lack of a denominator with which to calculate rates. In the absence of a denominator, RCP prescription data was utilized in the estimation of AE, SAE and cardiac AE rates. As a result, the adverse event reporting rate data provided may suffer from measurement bias. In addition, given the retrospective nature of this analysis, the potential impact of the implementation of the patient informed consent requirement following the November 2010 risk communication on changes in adverse event reporting behaviour of RCP recipients to GSK’s AE database, in comparison with their reporting behaviours prior to the implementation of the patient informed consent requirement, is unknown.

In the context of type 2 diabetes mellitus, there is an established increased risk of major cardiovascular complications and mortality, in comparison with people without diabetes mellitus [26, 27]. Results from an analysis of all-cause mortality among individuals with and without diabetes in the Framingham Heart study found that mortality rates are approximately twofold higher amongst individuals with diabetes mellitus compared to individuals without diabetes mellitus [26]. Similarly, the findings of collaborative meta-analysis of 102 prospective studies reported a twofold higher risk for a wide range of vascular diseases in people with diabetes in comparison with those without diabetes mellitus [27].

Consistent with RECORDs findings, the results of an analysis of RCP use and cardiovascular outcomes in the Veteran Affairs Diabetes Trial found that the use of rosiglitazone was associated with decreased risk of the primary cardiovascular composite outcome (myocardial infarction, cardiovascular death, stroke, congestive heart failure, invasive revascularization, inoperable coronary artery disease and amputation for ischemia) and cardiovascular death, and did not lead to a higher risk of myocardial infarction [28]. Similarly, an evaluation of patients with type 2 diabetes mellitus in the REACH registry found the use of thiazolidinediones was not associated with increased incidence of major cardiovascular events [29]. The results of the BARI 2D trial also support the RECORD findings [30].

## Conclusion

The results of this analysis show a substantial decrease in rosiglitazone utilization over the January 2011 to December 2012 period. A total of 32 adverse events in Canadian rosiglitazone recipients were reported to GSK’s global adverse event database during this time period with an average reporting rate of 10.8 events per 100,000 patients. Given the limitations of the methodology employed to evaluate reporting rates of adverse events, as well as other noted factors potentially impacting the reporting rate, the significance of this finding is limited.

## Abbreviations

AE: adverse events
aHR: adjusted hazard ratio
BARI 2D: bypass angioplasty revascularization investigation 2 diabetes trial
CAE: cardiac adverse events
CI: confidence interval
DCRI: Duke Clinical Research Institute
FDA: Food and Drug Administration
GSK: GlaxoSmithKline
RCP: rosiglitazone-containing product
REACH: REduction of Atherothrombosis for Continued Health
RECORD: Rosiglitazone Evaluated for Cardiovascular Outcomes and Regulation of Glycemia in Diabetes
REMS: risk evaluation and mitigation strategy
SAE: serious adverse events
US: United States
